# Association between kinesiophobia and bone mineral density in hospitalized older adults with osteoporosis: a cross-sectional study

**DOI:** 10.3389/fendo.2026.1731122

**Published:** 2026-02-03

**Authors:** MengPing Tian, WanQiu Lv, XinLing Miao, Cheng Chen, Jian Xiong, Jiali Zhang

**Affiliations:** 1 School of Basic Medical Sciences & School of Nursing, Chengdu University, Chengdu, China; 2 Clinical Medical College & Affiliated Hospital of Chengdu University, Chengdu, China

**Keywords:** aged, bone density, cross-sectional studies, fear of movement, osteoporosis

## Abstract

**Objective:**

To examine the association between kinesiophobia (fear of movement) and lumbar spine Bone mineral density (BMD) among hospitalized older adults with osteoporosis.

**Methods:**

This cross-sectional study included 246 hospitalized adults aged 60 years or older with osteoporosis who were admitted to orthopedic wards at the Affiliated Hospital of Chengdu University between August and October 2025. Kinesiophobia was assessed using the 17-item Tampa Scale for Kinesiophobia (TSK-17), with scores ≥37 indicating the presence of kinesiophobia. The primary outcome, volumetric trabecular bone mineral density (BMD), was measured at the lumbar spine (L1–L4) using quantitative computed tomography (QCT). Spearman correlation and multivariable linear regression analyses were performed to evaluate factors associated with BMD.

**Results:**

Among 246 participants (53 men,193 women),186 (75.6%) exhibited kinesiophobia. CT-measured BMD was significantly lower in participants with kinesiophobia (p < 0.001). Kinesiophobia showed a moderate negative correlation with BMD (ρ = –0.286, *p* < 0.001), while age demonstrated a strong negative correlation (ρ = –0.443, *p* < 0.001), and BMI a weak positive correlation (ρ = 0.148, *p* = 0.02). In multivariable analyses, kinesiophobia was independently associated with lower BMD (β, −8.897 mg/cm³; 95% CI, −13.715 to −4.079; *P* <.001). Older age was also associated with lower BMD (per 1-year increase: β, −0.516 mg/cm³; 95% CI, −0.694 to −0.338; *P* <.001). The overall model explained 20.1% of the variance in BMD.

**Conclusion:**

Among hospitalized older adults with osteoporosis, kinesiophobia was independently associated with lower bone mineral density. These findings underscore the clinical relevance of assessing and addressing fear of movement as part of comprehensive osteoporosis care in hospitalized older adults.

## 1 Introduction

Osteoporosis (OP) is a systemic skeletal disorder characterized by low bone mass and deterioration of bone microarchitecture, leading to increased fragility and fracture susceptibility (1). As populations age globally, the prevalence of OP has risen markedly, particularly among older adults. A meta-analysis estimated that approximately 21.7% of older individuals worldwide have OP, with a prevalence of 35.3% among elderly women (2). OP-related fractures can cause pain, disability, and decreased quality of life, placing substantial socioeconomic burdens on healthcare systems (3, 4).

Regular physical activity and weight-bearing exercise are fundamental for maintaining bone metabolism and preserving bone mass in patients with OP (5, 6). However, engagement in such beneficial activities is often hindered in older adults by pain, functional limitations, and particularly, by the fear of movement —or kinesiophobia (7). Kinesiophobia is defined as an excessive, irrational fear of movement stemming from concerns about pain or reinjury (8).It can trigger a vicious cycle of activity avoidance, physical deconditioning, and progressive deterioration of musculoskeletal health (9), as conceptualized within the fear-avoidance model of pain-related behavior (10).

Evidence from Sweden suggests that the prevalence of kinesiophobia increases with age, with older individuals more likely to experience it than younger one (11). This condition often leads to movement avoidance, decreased activity levels, and a progressive decline in overall health status (12). Over time, such inactivity can significantly reduce mechanical loading on bones, contributing to a decline in Bone Mineral Density (BMD) and subsequently increasing fracture risk (13). Although previous studies have examined kinesiophobia in populations with chronic pain, cardiovascular disease, or post–joint replacement (14–17), its specific relationship with bone mineral density—a primary indicator of skeletal health—in patients with osteoporosis remains poorly understood.

This knowledge gap is especially pertinent in hospitalized older adults with osteoporosis, a vulnerable population in whom acute illness, pain, immobility, and heightened fracture risk perceptions may converge to exacerbate fear of movement (18, 19). Currently, the association between kinesiophobia and BMD in this specific clinical setting has not been characterized. Elucidating this link is crucial for developing targeted rehabilitation strategies. Therefore, this cross-sectional study aimed to investigate the magnitude and independence of the association between kinesiophobia and QCT-measured volumetric BMD in hospitalized older adults with osteoporosis.

## 2 Methods

### 2.1 Study design and participants

This cross-sectional study was conducted in the Departments of Orthopedics I and II at the Affiliated Hospital of Chengdu University between August and October 2025. Participants were recruited through convenience sampling. All participants were hospitalized for orthopedic conditions, primarily due to osteoporotic fractures or Osteoporotic bone pain, with a median hospital stay of 5 days. Among the included participants, 44.31% were admitted for acute osteoporotic fractures, and 55.69% for Osteoporotic bone pain or related complications. Ethical approval was obtained from the Institutional Review Board of the Affiliated Hospital of Chengdu University (Approval No: PJ2025-092-02), and all participants provided written informed consent.

Inclusion criteria:①age ≥60years;②a confirmed diagnosis of OP;③voluntary participation. Exclusion criteria were:① psychiatric disorders or severe communication difficulties;②cognitive impairment due to dementia, Parkinson disease, or similar conditions;③Terminal-stage malignant tumors or an expected survival period of less than 6 months;④Secondary osteoporosis caused by medications or other diseases.

### 2.2 Sample size calculation

A sample size calculation was performed using G*Power (version 3.1). The sample size estimation was based on a moderate expected correlation coefficient (r = 0.25). Using a two-tailed test with a significance level of 0.05 and a statistical power of 0.90, the software indicated that a minimum of 158 participants would be required to detect a significant effect. To account for a potential 15% rate of missing data or invalid questionnaires, the adjusted target sample size was increased to 182 participants. Ultimately, a total of 246 older adults with osteoporosis were enrolled, exceeding the required number and ensuring adequate statistical power for the analysis.

### 2.3 Data collection

Data were collected through structured questionnaires administered by trained nursing graduate students and supplemented by medical record review. Demographic variables included age, sex, BMI, smoking status, alcohol consumption, marital status, education level, and history of falls/fractures. Clinical variables included pain characteristics, comorbidities (hypertension, diabetes, cardiovascular disease). BMI was categorized as underweight (<18.5 kg/m²), normal (18.5-23.9 kg/m²), overweight (24.0-27.9 kg/m²), and obese (≥28.0 kg/m²) based on criteria for Chinese adults. Bone mineral density was measured as volumetric trabecular BMD at the lumbar spine (L1–L4) using quantitative computed tomography (QCT), with results expressed in mg/cm³. QCT was chosen for its ability to evaluate volumetric trabecular bone density and reduce the confounding effects of degenerative changes common in older adults (20).

### 2.4 Assessment of kinesiophobia

The level of kinesiophobia was assessed using the Tampa Scale for Kinesiophobia (TSK), one of the most widely used tools internationally for evaluating fear of movement or reinjury. Originally developed by Kori et al. in 1990, the TSK consists of 17 items rated on a 4-point Likert scale (1 = strongly disagree to 4 = strongly agree), with items 4, 8, 12, and 16 reverse scored. Total scores range from 17 to 68, a score of 37 or higher indicates kinesiophobia. The threshold commonly used and validated in studies involving older adults and chronic pain populations (21, 22). In this study, we used the Simplified Chinese version of the TSK translated and culturally adapted by Hu et al (23). This version showed a Cronbach’s alpha coefficient of 0.778 and a test-retest reliability of 0.86.

### 2.5 Statistical analysis

All analyses were performed using SPSS Statistics (Version 27.0) and R software (version 4.2.2). Descriptive statistics were computed for all variables, with categorical data presented as frequencies (n) and continuous data as means ± standard deviations (x̄ ± SD). Group differences in BMD were assessed using independent-samples t-tests or one-way analysis of variance (ANOVA), as appropriate. Relationships between variables and BMD were evaluated via Spearman’s rank correlation analysis. Variables demonstrating a univariate association with BMD (*p* < 0.05) were entered into a multiple linear regression model. Regression results included unstandardized (B) and standardized (β) coefficients, t-values, p-values, and 95% confidence intervals (CI). Model fit was evaluated using R² and adjusted R², with overall significance determined by the F-test. Multicollinearity was examined via variance inflation factors (VIF), with VIF < 10 and tolerance > 0.1 indicating acceptable levels. BMI and Age were treated as a continuous variable in regression analyses to preserve statistical information and avoid bias associated with arbitrary categorization.

## 3 Results

**Figure 1:**
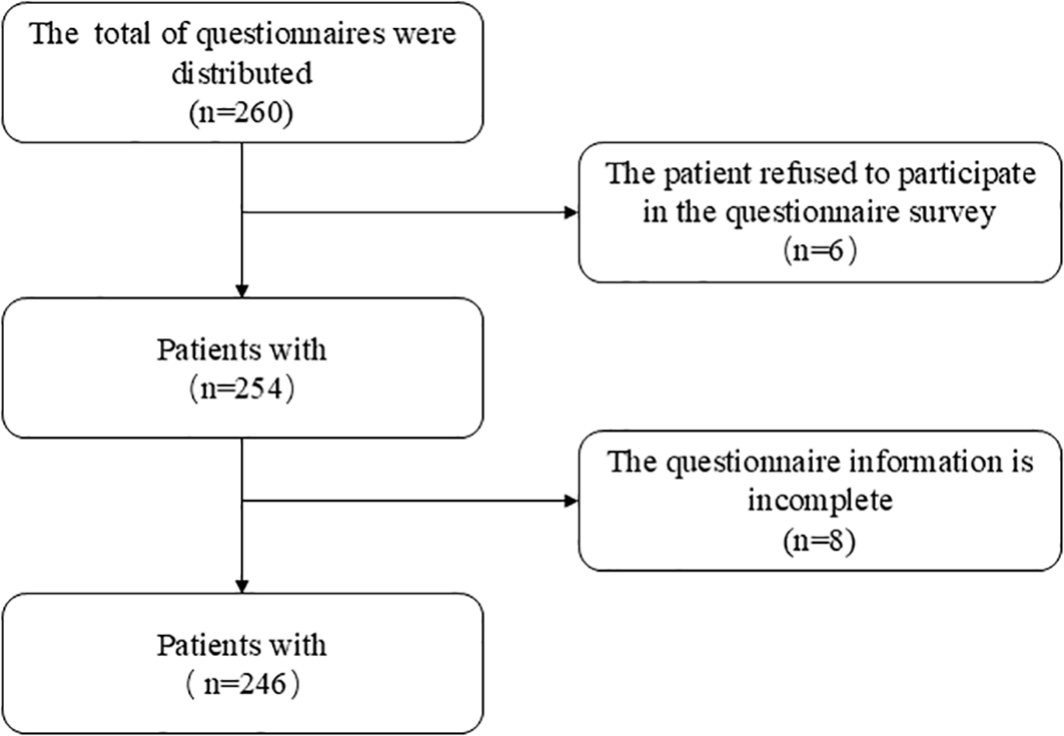
Flow diagram for the selection of participants included in the present analysis.

**Figure 2:**
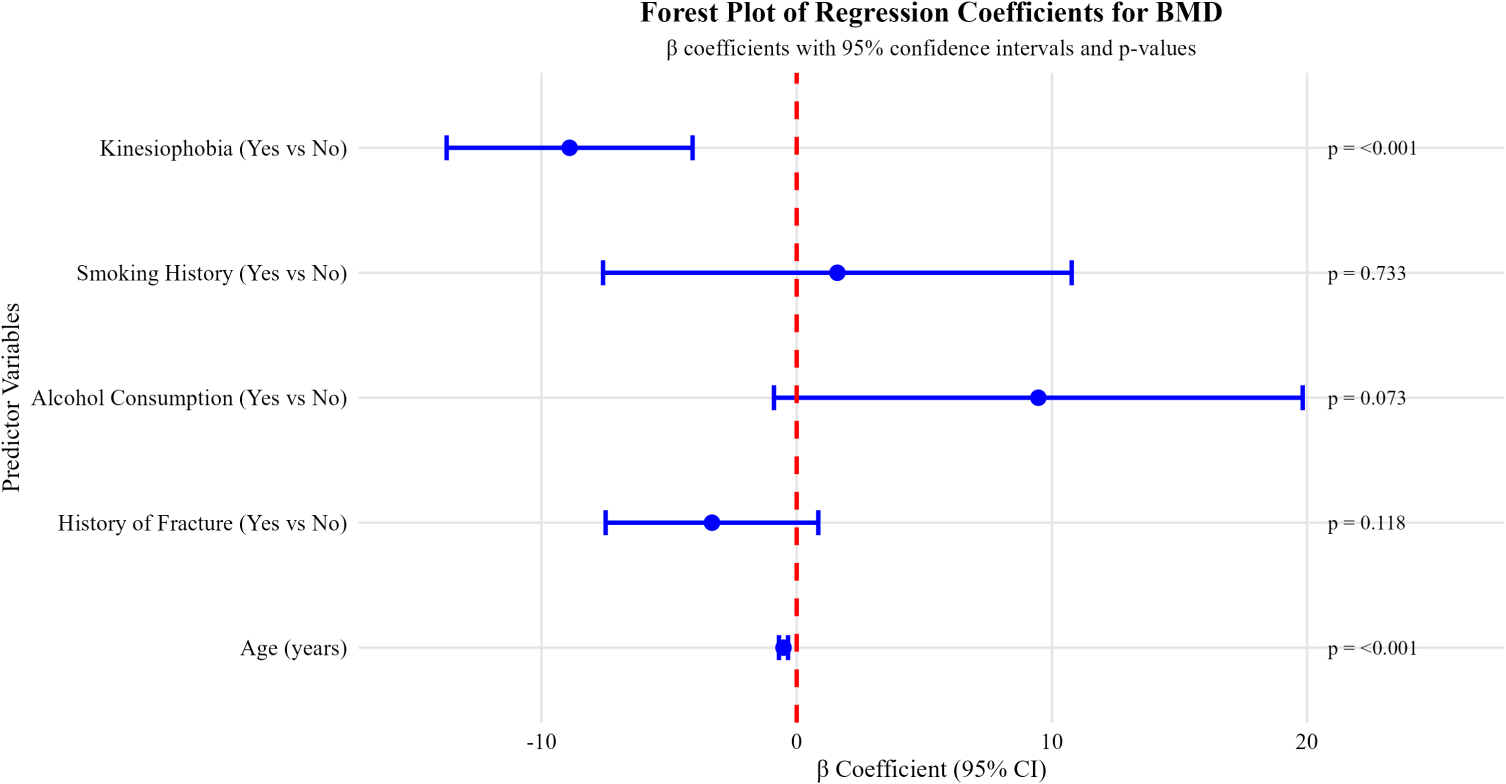
Comparison of BMD by categorical variables with statistically significant differences. Boxplots depict the distribution of BMD values (mg/cm³) measured by quantitative computed tomography. The central line within each box represents the median, the box boundaries indicate the interquartile range (25th to 75th percentile), and whiskers extend to 1.5 times the interquartile range. Individual data points are overlaid. Group comparisons were performed using independent two-sample t-tests.

Of the 260 patients initially screened, 14 were excluded based on predefined eligibility criteria. A total of 246 hospitalized older adults with osteoporosis were included in the final analysis (**Figure 1**). The median length of hospital stay was 5 days. A total of 246 hospitalized patients with osteoporosis were included in the study, comprising 53 males (21.5%) and 193 females (78.5%). Among them, 186 patients (75.6%) were identified as having kinesiophobia, while 60 patients (24.4%) did not exhibit kinesiophobia. The mean BMD in patients aged ≤74 years (n = 130) was 59.39 ± 14.69, which was significantly higher than that in patients aged >74 years (n = 116), whose mean BMD was 44.08 ± 17.61 (*P* < 0.001). Patients with a history of smoking or alcohol consumption had significantly higher BMD compared to those without such histories (*P* = 0.017 and *P* < 0.001, respectively). In contrast, patients with a history of fractures or with kinesiophobia had significantly lower BMD than those without these conditions (*P* = 0.005 and *P* < 0.001, respectively). No significant differences in BMD were observed in relation to sex, marital status, educational level, BMI category, comorbidities, presence of chronic pain, history of falls, or pain site and intensity *(P* > 0.05) (**Table 1**). Differences in BMD across categorical variables with statistically significant differences are shown in **Figure 2**.

**Table 1:** Comparison of BMD across demographic and clinical variables (Mean ± SD).

Variables	Groups	N	BMD (Mean ± SD)	t/F	P-value
Gender	Male	53	55.49 ± 14.97	1.727	0.087
	Female	193	51.26 ± 18.47		
Age group (years)	≤ 74	130	59.39±14.69	0.029	<0.001^*^
	>74	116	44.08±17.61		
Smoking history	Yes	22	59.68 ± 14.10	-2.547	0.017^*^
	No	224	51.43 ± 18.01		
Alcohol consumption	Yes	17	63.39 ± 10.13	-4.417	<0.001^*^
	No	229	51.34 ± 18.01		
History of falls	Yes	123	50.52 ± 17.91	1.454	0.147
	No	123	53.82 ± 17.67		
History of fractures	Yes	109	48.69 ± 17.51	2.76	0.005^*^
	No	137	54.93 ± 17.66		
Comorbidities (Any)	Yes	128	50.03 ± 17.83	0.228	0.728
	No	118	54.49 ± 17.61		
Kinesiophobia	Yes	186	50.00 ± 17.94	3.433	<0.001^*^
	No	60	58.89 ± 15.79		
Marital status	Single	5	46.24 ± 21.19	0.83	0.478
	Married	188	53.14 ± 17.43		
	Widowed	45	49.20 ± 19.24		
	Divorced	8	49.79 ± 17.80		
Education level	Middle school or below	214	52.61 ± 17.94	0.562	0.571
	High school	16	50.19 ± 15.14		
	College or above	16	48.19 ± 19.21		
BMI category	Underweight	20	51.01 ± 17.80	1.025	0.382
	Normal	114	50.88 ± 18.41		
	Overweight	79	52.32 ± 16.03		
	Obese	33	56.97 ± 19.80		
Pain site	No pain	109	52.75 ± 16.69	1.12	0.341
	Trunk	100	51.25 ± 18.54		
	Upper limbs	10	44.72 ± 23.76		
	Lower limbs	27	55.97 ± 17.13		
Pain severity	None	109	52.75 ± 16.69	0.607	0.611
	Mild	91	50.89 ± 18.23		
	Moderate	37	52.00 ± 18.75		
	Severe	9	58.79 ± 24.06		

BMD, Bone Mineral Density; BMI, Body Mass Index. P-values were calculated using independent t-tests or one-way ANOVA as appropriate; *P < 0.05.

Comorbidities (Any) refers to the presence of one or more of the following conditions: hypertension, diabetes, or cardiovascular disease.

BMD was measured at the lumbar spine (L1–L4) using quantitative computed tomography (QCT).

**Figure 3:**
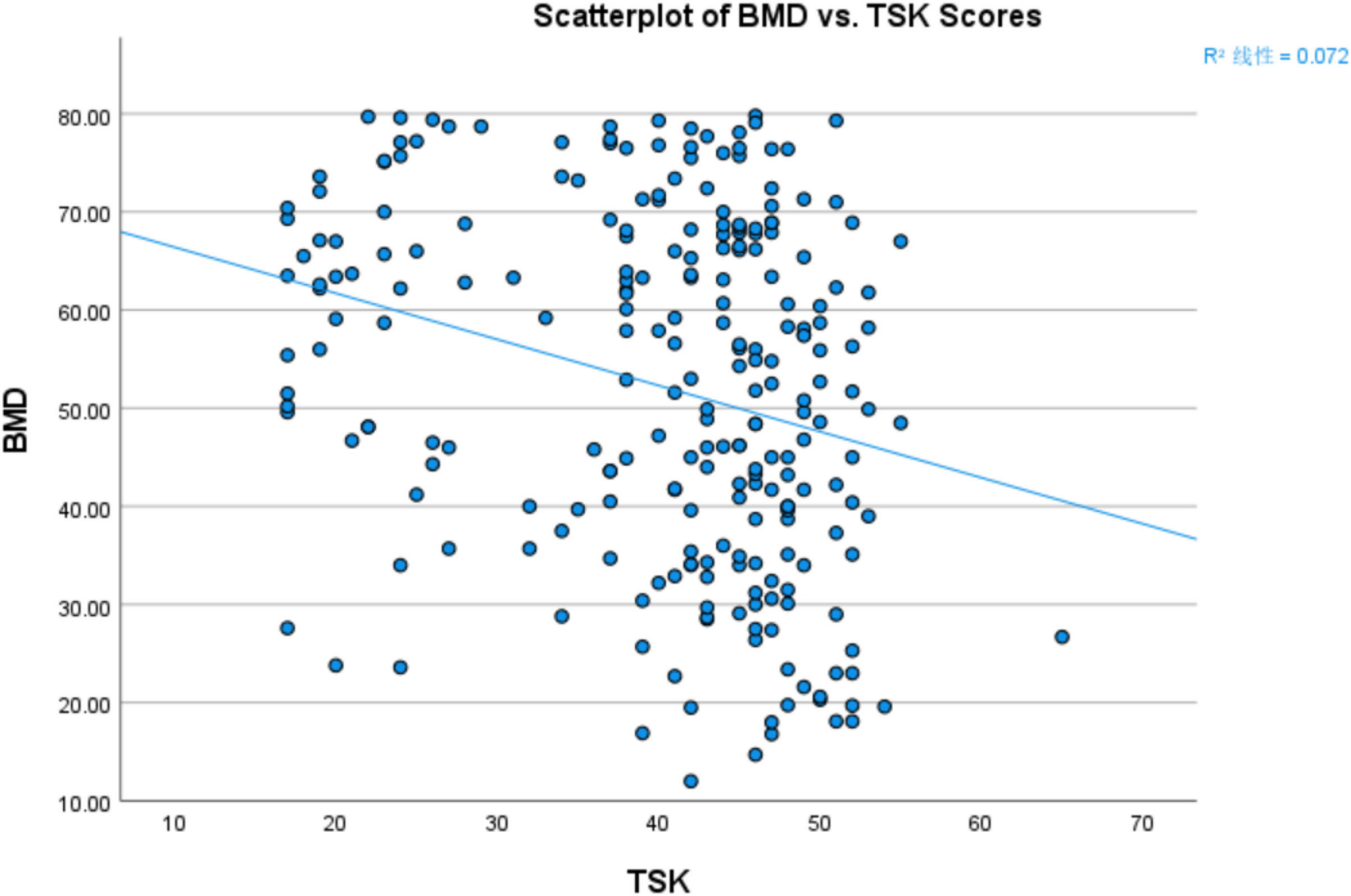
Scatterplot showing the correlation between BMD and kinesiophobia (TSK).

Given that the variables did not conform to a normal distribution, Spearman’s rank correlation analysis was employed to examine the associations between BMD and relevant continuous or ordinal variables (**Table 2**). The results showed that BMD was moderately negatively correlated with kinesiophobia (ρ = –0.286, *p* < 0.001), weakly positively correlated with BMI (ρ = 0.148, *p* = 0.02), and strongly negatively correlated with age (ρ = –0.443, *p* < 0.001). However, no significant correlations were observed between BMD and pain severity (ρ = 0.011, *p* = 0.867), education level (ρ = –0.068, *p* = 0.287), or marital status (ρ = –0.064, *p* = 0.318). A scatterplot was generated to visually illustrate the relationship between BMD and kinesiophobia, which was the primary focus of this analysis (**Figure 3**).

**Table 2:** Spearman’s correlation between BMD and related variables.

Variables	Spearman’s ρ	P-value
TSK	–0.286	<0.001
BMI	0.148	0.02
Age	–0.443	<0.001
Pain severity	0.011	0.867
Education level	-0.068	0.287
Marital status	-0.064	0.318

**Figure 4:**
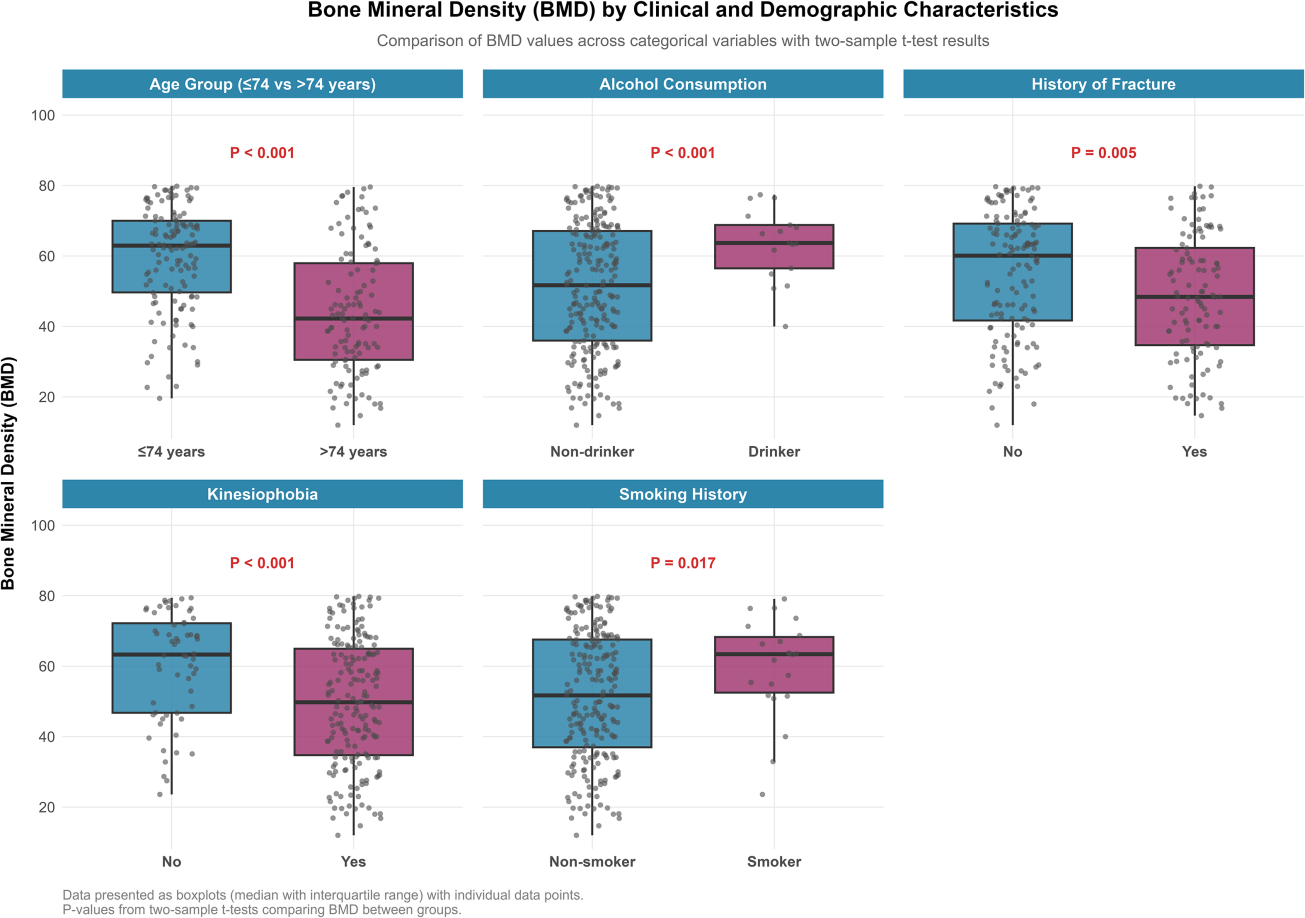
Forest plot of factors associated with BMD from multivariable linear regression analysis. Unstandardized regression coefficients (β) with their corresponding 95% confidence intervals are shown for each predictor included in the final model. The analysis adjusted for all other variables presented. The vertical dashed line at β = 0 represents the null value (no association). Predictors whose confidence intervals do not cross this line are considered statistically significant independent factors. Age was analyzed as a continuous variable (per year increase).

In multivariable linear regression analysis, kinesiophobia was independently associated with lower BMD, with an average decrease of 8.90 mg/cm³ compared to those without kinesiophobia (β, −8.897 mg/cm³; 95% CI, −13.715 to −4.079; *P* <.001). Older age was also independently associated with lower BMD (per 1-year increase: β, −0.516 mg/cm³; 95% CI, −0.694 to −0.338; *P* <.001). The independent associations identified by the multivariable linear regression model are presented in **Table 3** and illustrated in the forest plot (**Figure 4**). Smoking history, alcohol consumption, and history of fracture were not significantly associated with BMD after adjustment. The overall model explained 20.1% of the variance in BMD (**Table 4**).

**Table 3:** Regression coefficients for BMD predictors.

Predictor	β (95% CI)	P Value	VIF
Kinesiophobia	-8.897 (-13.715 to -4.079)	<0.001	1.07
Smoking History	1.595 (-7.587 to 10.776)	0.733	1.71
Alcohol Consumption	9.469 (-0.890 to 19.828)	0.073	1.72
History of Fracture	-3.319 (-7.486 to 0.847)	0.118	1.07
Age (years)	-0.516 (-0.694 to -0.338)	<0.001	1.01

Values are presented as β coefficient with 95% confidence interval.

BMD, Bone Mineral Density; VIF, Variance Inflation Factor.

**Table 4:** Model summary.

Statistic	Value
R	0.466
R²	0.217
Adjusted R²	0.201
SE	15.939
F	F(5, 240) = 13.31
P	<0.001

## 4 Discussion

In this cross-sectional study of hospitalized older adults with osteoporosis, kinesiophobia was independently associated with lower volumetric BMD. This association remained statistically significant even after adjusting for age and other clinical factors in a multivariable linear regression model.

Our results indicate a weak positive correlation between BMI and BMD (ρ = 0.148, p = 0.02), suggesting that higher BMI may be associated with increased BMD. A study in the United States found that for each unit increase in BMI, BMD increased by 0.0082 g/cm² (*p* < 0.001) (24). Similarly, a study from Taiwan reported that higher BMI categories were associated with lower osteoporosis prevalence and a reduced risk of developing osteoporosis over time (25). Additionally, some studies have suggested that maintaining a BMI in the slightly overweight range (around 26 kg/m²) may reduce other adverse effects while achieving optimal BMD (26). Nevertheless, despite this modest protective association, kinesiophobia remained independently associated with lower BMD after adjustment, suggesting that fear-related activity avoidance may exert detrimental skeletal effects beyond those explained by body mass alone.

Our study also found a significant negative correlation between age and BMD (ρ = –0.443, *p* < 0.001), meaning that BMD decreases as age increases. As individuals age, bone metabolism gradually slows down, and bone resorption intensifies, leading to a decrease in BMD. This decline is particularly pronounced in the elderly population. Previous studies have shown that as age increases, especially after women reach menopause, the decline in BMD becomes more significant (27). Additionally, the activity level of the elderly is often lower, and a sedentary lifestyle may accelerate the decline in BMD (28).Therefore, age is not only a natural factor in the development of osteoporosis but also influences bone health through interactions with lifestyle and hormonal changes.

Several interrelated behavioral and biological mechanisms may explain the observed association between kinesiophobia and lower BMD. From a behavioral perspective, the fear-avoidance model provides a well-established framework (10): excessive fear of movement or reinjury can lead to activity avoidance, resulting in reduced mechanical loading on the skeleton, a key osteogenic stimulus (29). In individuals with osteoporosis, the diagnosis itself may heighten fear of falling and fracture, which can both drive and reinforce kinesiophobia, creating a self-perpetuating cycle of inactivity and skeletal deconditioning (30).

From a biological perspective, chronic psychological stress associated with kinesiophobia may dysregulate the hypothalamic–pituitary–adrenal axis, leading to sustained elevations in cortisol levels. Elevated cortisol has been shown to suppress osteoblast differentiation and promote bone resorption, thereby creating a hormonal environment unfavorable for bone maintenance (31, 32). In the context of osteoporosis, fear of falling and fracture may therefore function as both a precipitating and reinforcing factor for kinesiophobia, further accelerating bone density decline through combined behavioral and neuroendocrine pathways (13, 33).

Thus, kinesiophobia is associated with bone loss through a synergistic interplay of behavioral avoidance and stress-mediated biological pathways. The inpatient environment of our study cohort provides additional context for interpreting these findings. The inpatient setting itself—characterized by acute pain, restricted mobility, and an unfamiliar environment—may act as a catalyst, heightening perceptions of vulnerability and fracture risk, thereby intensifying kinesiophobia (19). This setting-specific amplification highlights hospitalization as a critical window for early identification and targeted management of fear of movement. Addressing kinesiophobia during hospital stays may influence not only short-term functional recovery but also longer-term bone health trajectories (34).

### Limitations

This study has several limitations. First, as a cross-sectional analysis, causal inference between kinesiophobia and BMD cannot be established. Second, although we adjusted for key demographic and clinical variables, residual confounding may persist because factors such as calcium and vitamin D supplementation, use of anti-osteoporotic medications, objectively measured physical activity levels, and detailed hormonal profiles were not comprehensively assessed. These factors could influence both kinesiophobia (e.g., through pain perception) and BMD, and their absence may have led to residual confounding Third, the single-center design and convenience sampling may limit generalizability.

## 5 Conclusion

In conclusion, this study demonstrates that kinesiophobia is a significant and independent factor associated with reduced BMD in hospitalized older adults with OP. These findings highlight the critical need to integrate psychological assessment and intervention into routine OP care. Early screening for kinesiophobia, followed by tailored interventions—such as cognitive-behavioral therapy to address fear, combined with supervised, progressive exercise training—may help mitigate fear of movement, promote engagement in bone-strengthening activities, and ultimately reduce fracture risk in this vulnerable population.

## Data Availability

The original contributions presented in the study are included in the article/supplementary material. Further inquiries can be directed to the corresponding authors.
